# Supplementary material for: Splice-Junction-Based Mapping of Alternative Isoforms in the Human Proteome
Source: Cell Rep. Author manuscript; Available in PMC 2020 Jan 15. (PMC6961840; doi:10.1016/j.celrep.2019.11.026)
Supplement: 2 [file NIHMS1546469-supplement-2.zip › DF1/PXD010154/AdrenalGland/AdrenalGland_21_PDXDC1_QEVEATAGLLYVDDPNWSGIGVVSFRNSGR.pdf]

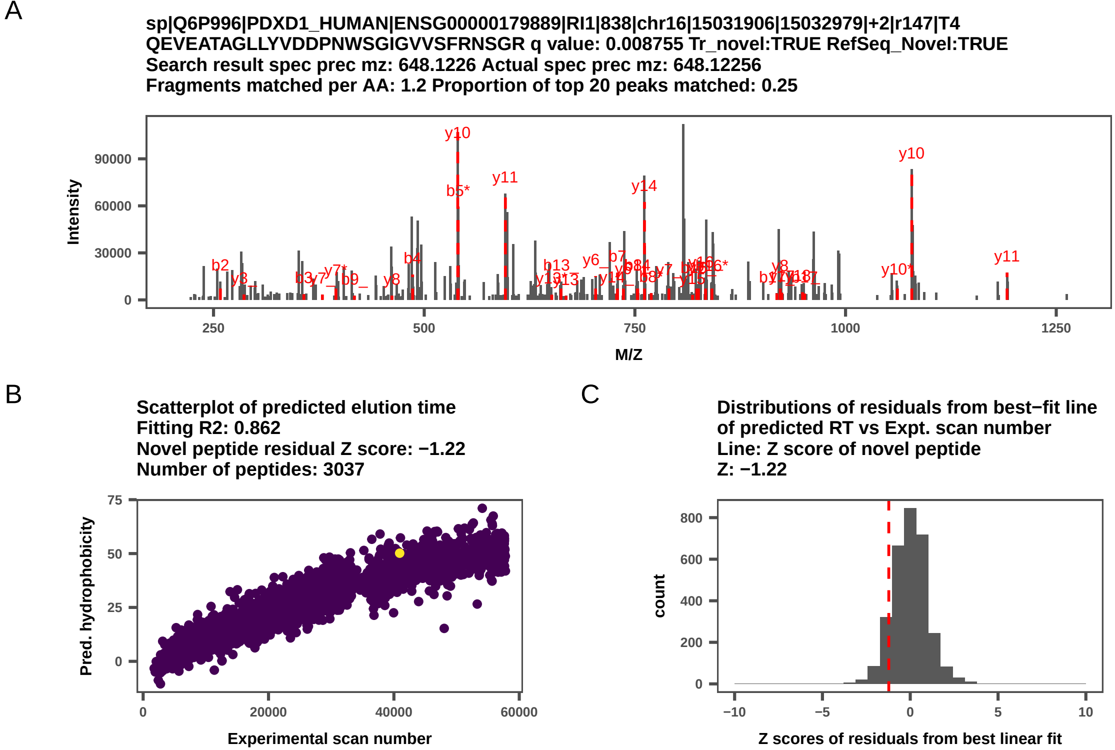
A
sp|Q6P996|PDXD1_HUMAN|ENSG00000179889|RI1|838|chr16|15031906|15032979|+2|r147|T4
QEVEATAGLLYVDDPNWSGIGVVSFRNSGR q value: 0.008755 Tr_novel:TRUE RefSeq_Novel:TRUE
Search result spec prec mz: 648.1226 Actual spec prec mz: 648.12256
Fragments matched per AA: 1.2 Proportion of top 20 peaks matched: 0.25
Intensity
90000
60000
30000
0
250
500
750
1000
1250
M/Z
y10
y11
y14
y10
y11
B
Scatterplot of predicted elution time
Fitting R2: 0.862
Novel peptide residual Z score: −1.22
Number of peptides: 3037
Pred. hydrophobicity
75
50
25
0
0
20000
40000
60000
Experimental scan number
C
Distributions of residuals from best−fit line
of predicted RT vs Expt. scan number
Line: Z score of novel peptide
Z: −1.22
count
800
600
400
200
0
−10
−5
0
5
10
Z scores of residuals from best linear fit
